# Lemnaceae and Orontiaceae Are Phylogenetically and Morphologically Distinct from Araceae

**DOI:** 10.3390/plants10122639

**Published:** 2021-11-30

**Authors:** Nicholas P. Tippery, Donald H. Les, Klaus J. Appenroth, K. Sowjanya Sree, Daniel J. Crawford, Manuela Bog

**Affiliations:** 1 Department of Biological Sciences, University of Wisconsin-Whitewater, Whitewater, WI 53190, USA; tipperyn@uww.edu; 2 Department of Ecology and Evolutionary Biology, University of Connecticut, Storrs, CT 06269, USA; don.les@uconn.edu; 3 Matthias Schleiden Institute—Plant Physiology, University of Jena, D-07743 Jena, Germany; klaus.appenroth@uni-jena.de; 4 Department of Environmental Science, Central University of Kerala, Periye 671320, India; ksowsree@gmail.com or; 5 Department of Ecology and Evolutionary Biology, University of Kansas, Lawrence, KS 66045, USA; dcrawfor@ku.edu; 6 Natural History Museum and Biodiversity Research Center, University of Kansas, Lawrence, KS 66045, USA; 7 Institute of Botany and Landscape Ecology, University of Greifswald, D-17489 Greifswald, Germany

**Keywords:** aquatic plants, Araceae, duckweeds, Lemnoideae, molecular phylogenetics, taxonomy

## Abstract

Duckweeds comprise a distinctive clade of pleustophytic monocots that traditionally has been classified as the family Lemnaceae. However, molecular evidence has called into question their phylogenetic independence, with some authors asserting instead that duckweeds should be reclassified as subfamily Lemnoideae of an expanded family Araceae. Although a close phylogenetic relationship of duckweeds with traditional Araceae has been supported by multiple studies, the taxonomic disposition of duckweeds must be evaluated more critically to promote nomenclatural stability and utility. Subsuming duckweeds as a morphologically incongruent lineage of Araceae effectively eliminates the family category of Lemnaceae that has been widely used for many years. Instead, we suggest that Araceae subfamily Orontioideae should be restored to family status as Orontiaceae, which thereby would enable the recognition of three morphologically and phylogenetically distinct lineages: Araceae, Lemnaceae, and Orontiaceae.

## 1. Introduction

### 1.1. Taxonomic History of Araceae

**Figure 1:**
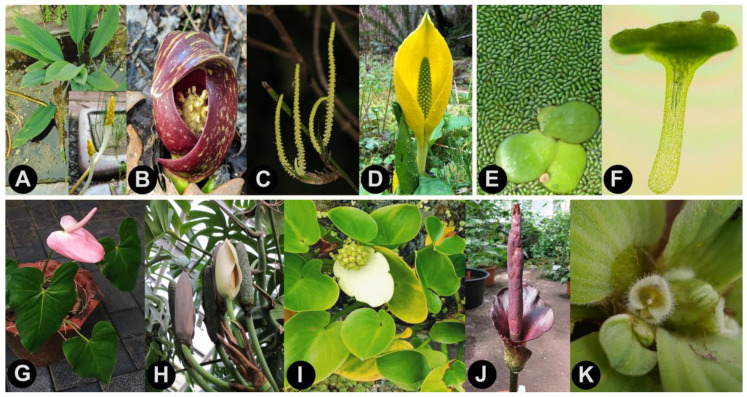
Species of Arales, divided among the family-level categories that are described herein: Orontiaceae (**A**–**D**), Lemnaceae (**E**,**F**), and Araceae (**G**–**K**). (**A**) Vegetative growth and inflorescence (inset) of *Orontium aquaticum* (photo credit: Wolfgang Pomper), (**B**) *Symplocarpus foetidus* (photo credit: Claire O’Neill), (**C**) Inflorescence of *Gymnostachys anceps* (photo credit: Leith Woodall), (**D**) *Lysichiton americanus* with inflorescence (photo credit: Ryan Kurtz), (**E**) Fronds of *Spirodela polyrhiza* surrounded by *Wolffia globosa*, (**F**) Light micrograph of *Wolffia microscopica* bearing the floral organs on the dorsal side (anther lobes seen on the top), (**G**) *Anthurium andraeanum* with inflorescence, (**H**) *Monstera deliciosa* with inflorescence (photo credit: Wolfgang Pomper), (**I**) *Calla palustris* with inflorescence, (**J**) *Amorphophallus konjac* with inflorescence (photo credit: Wolfgang Pomper), (**K**) Inflorescence of *Pistia stratiotes* (photo credit: Bo-Fu Sun).

**Figure 2:**
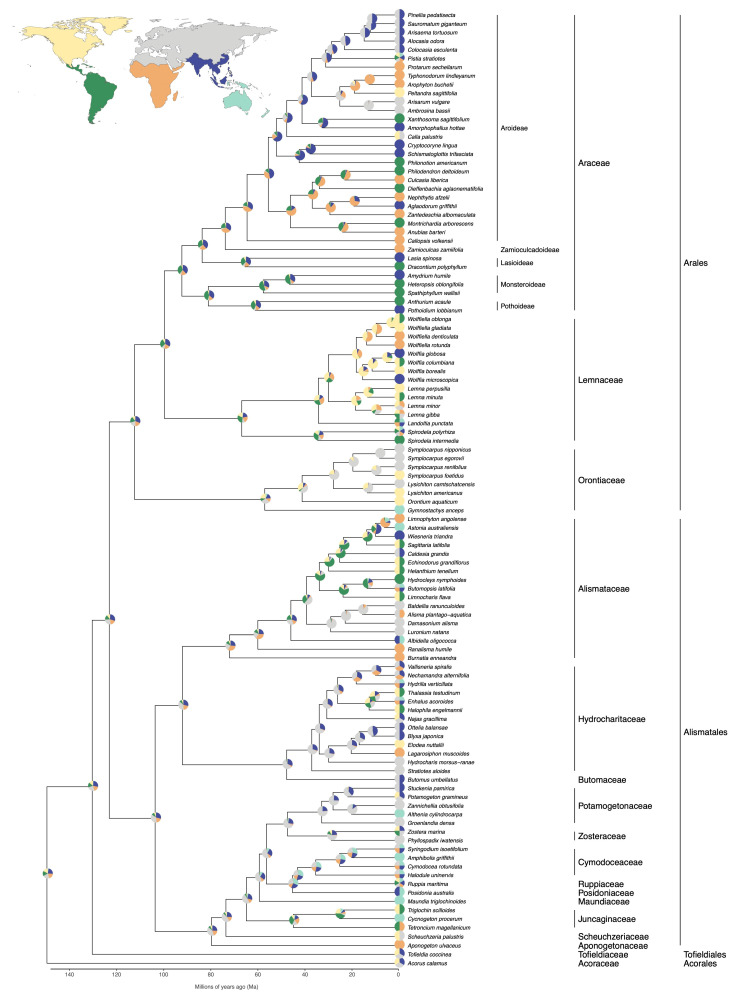
Phylogeny of Arales, constructed using combined DNA sequence data from five plastid regions (*matK*, *ndhF*, *rbcL*, *rps16*, and *trnL-F*). The tree represents a maximum likelihood tree, constructed in IQ-TREE [18] using default settings. Taxa were trimmed to include only one representative species for most genera. Branch lengths were adjusted to be ultrametric using the *chronopl* function in the R package *ape* [19], and the x-axis was scaled to approximate node ages that were reported previously [20]. Ancestral biogeography was reconstructed using the *ace* function in the *ape* package, to estimate the likelihood of an ancestor occupying one or more of the biogeographical realms indicated by the colored regions in the inset map. The ancestral likelihood values are shown as pie charts at the nodes of the phylogeny.

Araceae Juss. (aroids) are one of the larger angiosperm families, comprising around 5000 species that are distributed primarily in tropical latitudes [1,2]. These plants have a variety of identifying characteristics, including calcium oxalate crystals and tiny flowers that are borne on a distinctive spadix inflorescence [1] (Figure 1). The application of molecular data to angiosperm phylogenetic analyses has sparked greater confidence in angiosperm classification, and Araceae are no exception. Molecular data have validated several monophyletic subfamilies and enabled a richer interpretation of their morphological evolution [3,4,5,6]. Molecular data also produced a somewhat unexpected result, namely that duckweeds, long classified as the separate family Lemnaceae Martinov, nom. cons. [7,8], were descended from the same common ancestor as Araceae [3]. Because a number of molecular phylogenetic analyses have grouped duckweeds with Araceae, modern taxonomic treatments have begun to assign duckweeds to an aroid subfamily (Lemnoideae Engler) in order to preserve Araceae as monophyletic [3,4,5,6,9,10]. However, many of the same studies have shown Lemnaceae to be phylogenetically and morphologically distinct [5,11,12], with the duckweed lineage diverging around 104 Ma (Figure 2) [6]. Although the expansion of Araceae to include duckweeds is one solution to reconcile the phylogenetic observations, there also are other options that would allow the primary taxonomic categories to remain consistent with phylogenetic lineages; yet, these seem to have been given little consideration.

An alternative option that would preserve both Araceae and Lemnaceae as monophyletic would be to restore the araceous lineages of Gymnostachydoideae Bogner and Nicolson and Orontioideae Mayo, Bogner and Boyce collectively to the family level as Orontiaceae Bartl. These plants, often referred to as ‘proto-Araceae’, are the phylogenetic sibling lineage of duckweeds plus the remaining Araceae, having diverged around 122 Ma (Figure 2) [3,4,5,6]. For clarity, we will refer to the clade that includes Gymnostachydoideae, Lemnoideae, and Orontioideae as Araceae s.l. (*sensu lato*) (as suggested by APG [10]), and the clade of Araceae lacking these subfamilies (sometimes referred to as the ‘true Araceae’ [1,4,5,13] or the ‘core Araceae’ [14,15,16,17]) as Araceae s.s. (*sensu stricto*) (i.e., our recommended taxonomic disposition). We propose that taxa in Araceae s.l. should be divided among the established families Araceae s.s., Lemnaceae, and Orontiaceae. Besides being separated by ancient and long evolutionary branches, these families are morphologically divergent and well suited to a classification scheme that highlights their distinctness while similarly preserving more traditional morphological concepts for both groups.

**Figure 3:**
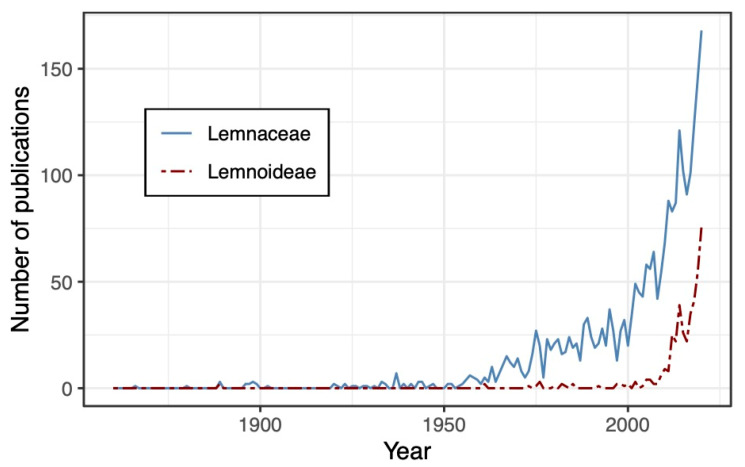
Usage of ‘Lemnaceae’ and ‘Lemnoideae’ in publications over time. Important events that may have influenced usage include the initial plastid data suggesting that duckweeds belong to the Araceae clade in 1995 [3] and the APG recommendation to include duckweeds in Araceae s.l., in 1998 [10]. Data were obtained from the Dimensions website (https://app.dimensions.ai/; accessed on 20 August 2021) by searching for each keyword anywhere in an article.

A great deal of diversity is contained in Araceae s.l., comprising morphological diversity in the extreme growth forms of duckweeds and terrestrial aroids, as well as evolutionary diversity in the large number of species and the ancient origin of the lineage. Some of the more prominent angiosperm and monocot phylogenetic studies have advocated for the Araceae s.l. circumscription, but a universal criterion does not exist for defining the boundaries of a plant family. We believe that the ultimate taxonomic disposition of aroids and duckweeds should integrate data from multiple disciplines and perspectives, and not be based simply on the opinions of either the broad-scale angiosperm phylogenetic community or the group of scientists who have devoted their careers to studying the traditional Araceae (i.e., Araceae s.s. plus Orontiaceae). Although it has been nearly 30 years since molecular data first suggested a close relationship between aroids and duckweeds [3], the usage of ‘Lemnaceae’ has remained quite prevalent in the literature (Figure 3). Thus, it remains necessary to provide an objective, equitable, and stable solution to the taxonomic disposition of these exciting and diverse angiosperms.

### 1.2. Orontiaceae

In several recent phylogenetic studies, Gymnostachydoideae (*Gymnostachys* R. Br.) and Orontioideae (*Lysichiton*, *Orontium* L., *Symplocarpus* Salisb. ex W. P. C. Barton) are considered as subfamilies of Araceae s.l. [4,5,6]. Araceae originally included *Orontium* [21], and eventually, the genera *Gymnostachys* [22], *Lysichiton* [23], and *Symplocarpus* [24] also were described within the aroids. Infrafamilial classifications of the aroids have varied considerably, but most have acknowledged the distinctness of the ‘proto-Araceae’ genera [25,26,27,28]. The category Orontiaceae, originally erected to accommodate several exceptional aroid genera [29], would be appropriate for encompassing the taxa currently designated as the subfamilies Gymnostachydoideae and Orontioideae. 

Prior to the advent of molecular systematics, the classification by Engler [26] closely approximated the current phylogenetic hypothesis by placing *Lysichiton*, *Orontium*, and *Symplocarpus* together (in Calloideae: Symplocarpeae), yet this classification also grouped *Gymnostachys* in a separate subfamily (Pothoideae: Acoreae) with *Acorus* L. Further analysis of morphological data led to the segregation of Acoraceae Martinov, while retaining *Gymnostachys* within Araceae [30]. Molecular data clearly have advanced our understanding of character evolution since that time, and the consistent phylogenetic placement of subfamilies Gymnostachydoideae and Orontioideae enables a more confident evaluation of their shared morphological characters [3,4,5,6,31].

Whereas the overall inflorescence morphology in *Lysichiton* and *Symplocarpus* resembles the spathe + spadix model of Araceae s.s., the inflorescences of *Gymnostachys* are branched, and those of *Gymnostachys* and *Orontium* lack a subtending spathe [26,32]. It should be noted also that the spadix inflorescence is not uniquely synapomorphic to aroids, with plants in the order Acorales Martinov producing a similar inflorescence [30,33]. Several features, such as stomata type and flavonoid profile, unite Orontioideae and distinguish this group from most other Araceae s.l. [28,34,35]. Shared features of Gymnostachydoideae and Orontioideae include seedlings with cataphylls [36], rhizomatous growth [28], inflorescences with uniformly hermaphroditic flowers [5], dimerous flowers (trimerous in *Orontium* [37]), pollen walls with ektexine [38,39], unilocular and uniovulate gynoecia (bilocular with 1–2 ovules per locule in *Lysichiton* [28]), orthotropous ovules (hemianatropous in *Orontium* [40]), and apical placentation (basal in *Orontium* [28,37]. Orontiodeae plants lack vessels entirely [41], and this feature has not been investigated in Gymnostachydoideae. Although some of the morphological features found in Gymnostachydoideae and Orontioideae also occur in various Araceae s.s. taxa, the combination of these features unites the ‘proto-Araceae’ (i.e., Orontiaceae according to our suggestion) reasonably well.

Just nine species are classified within the four Orontiaceae genera, and their distinctness extends beyond morphology and anatomy to include biogeography and habitat [31]. *Lysichiton*, *Orontium*, and *Symplocarpus* are denizens of temperate wetland or aquatic habitats in the northern hemisphere, which contrast with the tropical distributions and terrestrial habitats of most Araceae s.s. [42,43]. *Gymnostachys* grows in moist forest habitats in eastern Australia, another location where relatively few other Araceae s.s. are found [1]. Consequently, we believe that the phylogenetic, morphological, and ecological divergence of these genera is more than sufficient to justify their recognition as an independent family. Also, the taxonomic reassignment of ten ‘proto-Araceae’ species to Orontiaceae would be relatively minor, in sharp contrast with the migration of 36 duckweed species [44] from Lemnaceae into Araceae s.l., which became necessary upon acceptance of the APG classifications [10].

### 1.3. Lemnaceae

Duckweeds (Lemnaceae) are an aquatic monocot lineage that had been difficult to classify using morphological data, owing to their extreme reduction in size and complexity [11,45]. Morphological classifications have long supposed a close relationship between duckweeds and the araceous pleustophyte genus *Pistia* L. [26,46], yet the similar aquatic habits in these groups are convergent rather than synapomorphic [3]. Duckweeds are not particularly close relatives of Araceae s.s., with their common ancestor coalescing over 100 Ma, but their phylogenetic position as descendants from the shared ancestor of Araceae s.l. has caused them to lose their independent family designation [10]. Faced with molecular evidence that the two families were not reciprocally monophyletic [3], botanists soon developed a consensus that Araceae and Lemnaceae should be merged [10]. Although it makes evolutionary sense to infer the extreme morphological reduction of duckweeds from an araceous ancestor, nevertheless, the morphology of extant duckweeds cannot easily be equated with many features that are shared between Orontiaceae and Araceae s.s.

Duckweeds are diverse and ecologically unique, occupying the surface or subsurface layer of water bodies [11], reproducing extremely effectively through vegetative means [47,48,49], and even dispersing as whole-plant units by adhering to the bodies of aquatic fauna [50]. The uniqueness of the duckweed growth strategy has caused them to be regarded as a separate plant family for nearly the entire time since their discovery [51,52,53]. There are abundant features that distinguish Lemnaceae from both Araceae s.s. and Orontiaceae beyond the obvious reduction in morphological size and complexity. Duckweeds have unique ulcerate, spinose pollen [5,54], and there are numerous taxonomically informative characters that differentiate duckweed genera and species [11,55,56]. The category of Lemnaceae has been in use for two centuries [7,51,57], and importantly, there is extensive literature written by researchers who specialize on duckweeds.

Morphological and molecular analyses of Lemnaceae continue to validate this lineage as distinct and worthy of independent taxonomic recognition [12,15,44,58,59]. Whereas an initial molecular phylogenetic study showed duckweeds to be deeply nested within Araceae s.l. [3], subsequent and more thorough studies have consistently placed them as rather distantly related to Araceae s.s., with only Orontiaceae preventing them from being reciprocally monophyletic with the traditional Araceae (i.e., Araceae s.s. plus Orontiaceae) [4,5,6]. In more recent years, genome sequencing of several duckweed species has provided additional molecular and cytological data, with authors largely continuing to promote the taxonomic independence of Lemnaceae [60,61,62,63,64]. In the 20 years since the APG revision [10], the taxonomic term ‘Lemnaceae’ has been used consistently more than the term ‘Lemnoideae’ (Figure 3), and studies focusing specifically on duckweeds predominantly refer to them as an independent family [12,15,44,47,48,55,56,58,59]. It is reasonable to suggest that a taxonomic solution that preserves the nomenclature currently used by duckweed biologists would be preferable. 

### 1.4. Related Lineages and Ordinal Classification

According to widely accepted phylogenetic analyses of plastid data, the sister lineage to Araceae s.l. is diverse and comprises several wholly aquatic lineages such as Hydrocharitaceae and Potamogetonaceae [20,65]. More distantly related to these, the family Tofieldiaceae resolves as the sister lineage of the clade containing Araceae, Hydrocharitaceae, and Potamogetonaceae.

The most recent APG ordinal classification scheme [66] recognizes a single order, Alismatales Dumort. that includes the diverse families Araceae, Hydrocharitaceae, Potamogetonaceae, and Tofieldiaceae. Opinions differ regarding the appropriate ordinal classification of these lineages, however, and other authors prefer to divide the alismatid monocots among two or more orders. Several recent publications [17,67,68] recognize Arales Dumort. as separate from Alismatales and Tofieldiales Reveal & Zomlefer [69], and other authors [17,65,70] additionally consider Potamogetonales Dumort. (=Zosterales Nakai) to be distinct from Alismatales. To avoid confusion, we will refer to the expanded order comprising Alismataceae and Araceae as Alismatales s.l., and the more narrow-sense order, limited to include Alismataceae, Hydrocharitaceae, and related lineages (but not Araceae s.l.) as Alismatales s.s.

As advocated by the APG [66], the order Alismatales s.l. is equivalent to subclass Alismatidae Takht. [71] plus Araceae s.l. and Tofieldiaceae [10,20]. Prior to molecular phylogenetic studies, Araceae and Lemnaceae (along with Acoraceae) comprised a separate order, Arales [72]. With the expansion of Araceae, the ordinal category of Arales became synonymous with Araceae s.l. and fell out of favor [10]. Although the diverse families of Araceae s.s., Alismataceae, Lemnaceae, Orontiaceae, Tofieldiaceae, and others are indeed monophyletic [4,58,65,73], their common ancestor extends back to ca. 130 Ma, not long after the ca. 139 Ma crown age for all extant angiosperms [20]. (Note that estimates of ancient diversification events are bounded by considerable uncertainty. We will use the time scale established in Ref. [20], but a range of other estimates exist for alismatid monocots [14,68]. Regardless of the dating method used, the relative ages of phylogenetic nodes are consistent across studies that use plastid sequence data). The immense morphological and ecological diversity contained in this lineage is rather difficult to conceptualize as a single order. Instead, we propose that it would be simpler and clearer to differentiate four categories at the ordinal rank: Alismatales (crown age 100 Ma), Potamogetonales (103 Ma), Tofieldiales (100 Ma), and Arales (122 Ma) [6,20,74]. The crown ages of these lineages would then be closer to the average range of values for other angiosperm orders.

The practice of using the Alismatales category to contain such diverse lineages as Araceae, Potamogetonaceae, and Tofieldiaceae [10,66] makes it more difficult to refer specifically to distinct evolutionary units within monocots. A broader diversity of taxonomic categories would enable a more nuanced discussion of the diversity of alismatid monocots. To facilitate discussion, in this paper we will consider Arales to be synonymous with Araceae s.l. (as described above), and we will limit the Alismatales s.s. category to the clade containing Alismataceae, Hydrocharitaceae, and related lineages (Figure 2). We also will use the categories Acorales, Potamogetonales, and Tofieldiales, as appropriate. The ordinal classification scheme used in this paper is aligned with ordinal categories that were widely accepted prior to the undeniably influential APG publications [10,66].

### 1.5. Objectives

Aroids and duckweeds have been categorized inconsistently in the time since the first molecular phylogenetic analyses were conducted, with some authors preferring to sink duckweeds within Araceae s.l. and others preserving the more traditional Lemnaceae category. The debate about taxonomic categories has not necessarily considered all evidence in an objective approach that promotes nomenclatural stability, universal criteria for taxonomic boundaries, and morphological diagnostics. Therefore, we endeavored to evaluate the available morphological, phylogenetic, and other evidence in order to determine the most appropriate classification scheme for these diverse and economically important plants.

## 2. Molecular Phylogenetic Evidence

The phylogenetic relationships among Orontiaceae, Lemnaceae, and Araceae s.s. are widely accepted, but the comprehensive phylogenetic analyses to date have focused primarily on plastid data. After some initial uncertainty about the phylogenetic position of Lemnaceae, they now consistently resolve as a strongly supported clade that is sister to Araceae s.s., with Orontiaceae being sister to the clade of Araceae s.s. plus Lemnaceae. All three families are separated by substantial branch lengths and receive high statistical support. The phylogenetic relationship of Lemnaceae with Araceae and Orontiaceae has provided the foundation for classification schemes that prefer to lump all three families into one large Araceae s.l.

### 2.1. Plastid Molecular Data

Araceae s.s. comprise a large number of species with a most recent common ancestor that diversified roughly in the last 100 Ma [6]. Recent studies have used a combination of plastid sequence data from the more variable spacer and intron regions (e.g., *trnK* introns, *trnL-trnF* spacer) and more conserved protein-coding sequences (e.g., *matK*, *ndhF*, *rbcL*, and *rps16* genes) to investigate relationships among species [4,5,6]. These plastid regions have become the backbone of evidence for phylogenetic relationships in Araceae s.l., and the taxonomic sampling from these regions has been extensive [4,5]. Recent years have seen a rapid expansion of studies that compare whole plastid genomes (e.g., [75,76,77]), and while these provide a wealth of informative molecular data, the sampling remains limited in many cases.

We conducted updated phylogenetic analyses using plastid, mitochondrial, and nuclear DNA sequence data. The majority of sequence data used in this study were published previously, with some sequences newly generated to augment the taxon sampling for groups of interest (Supplementary Table S1). Phylogenetic trees were obtained by conducting maximum likelihood analyses using IQ-TREE version 1.6.12 [18,78] with integrated model selection. Five plastid regions were included: *matK*, *ndhF*, *rbcL*, *rps16*, and *trnL-trnF* [5,58,79,80], and the plastid data matrix was trimmed to include a maximum of four species per genus. Where possible, sequence data were obtained from complete plastid genome sequences, which are becoming increasingly available [75,76,81,82,83,84,85,86,87].

**Figure 4:**
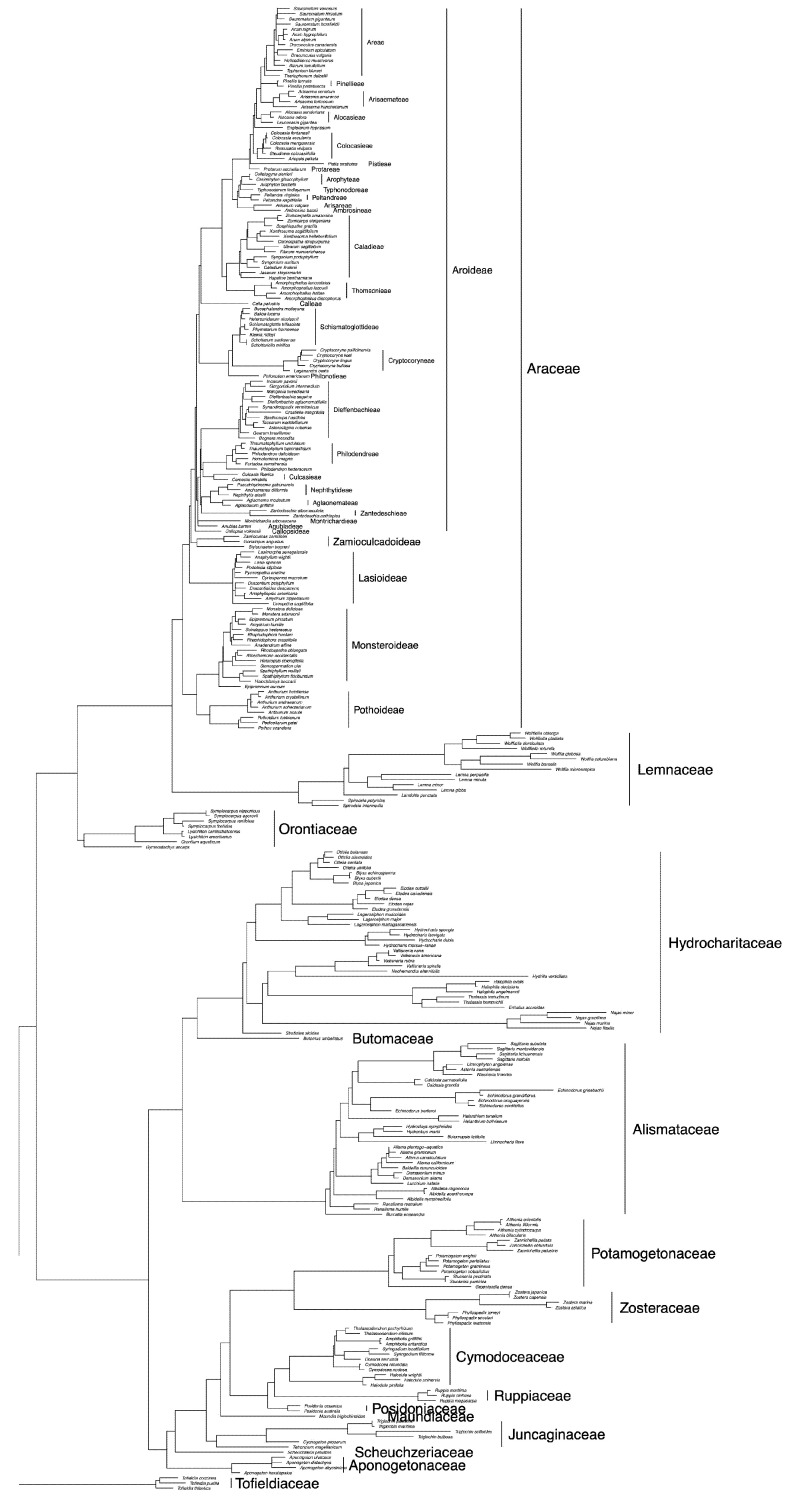
Phylogeny of Arales, constructed using combined DNA sequence data from five plastid regions (*matK*, *ndhF*, *rbcL*, *rps16*, and *trnL-F*), showing more complete taxon sampling from Arales genera. The tree represents a maximum likelihood tree, constructed in IQ-TREE [18] using default settings.

The plastid phylogeny corroborates prior evidence for the monophyly of orders Alismatales s.s., Arales, and Tofieldiales, as well as the families Orontiaceae, Lemnaceae, and Araceae s.s. (Figure 2). Araceae subfamilies also are monophyletic on the tree, and a more thoroughly sampled tree shows the tribal categories to be monophyletic as well (Figure 4). These relationships have been shown previously and are generally accepted [4,5,6]. Figure 2 represents an ultrametric tree, where branch lengths are approximately equal to time. Branch lengths on the maximum likelihood tree were transformed using the penalized likelihood method [88], employed in R version 4.1.1 [89] using the *chronopl* function in the *ape* package version 5.0 [19]. Besides the sequence data that support the phylogenetic relationships of Araceae s.s., Lemnaceae, and Orontiaceae, there are molecular patterns that also support the independence of these lineages. For example, a comparison of Arales genomes has identified duplications of the *rps15* and *ycf1* genes that are synapomorphic to Lemnaceae [75,85,87]. Additional patterns of this sort may be identified as more plastid genomes are sequenced across Arales.

### 2.2. Nuclear Molecular Data

In contrast to the relative abundance of plastid sequence data, far fewer studies in Arales have included nuclear DNA sequence data. The nuclear ribosomal internal transcribed spacer (nrITS) region commonly is used to reconstruct relationships among species, because the sequences evolve fairly quickly and can be sequenced with relative ease [90]. The nrITS region is situated between the large and evolutionarily conserved 18S and 26S ribosomal RNA genes, which themselves can be useful for determining larger-scale evolutionary patterns such as the relationships among families and orders [91,92]. Potential reasons to avoid nuclear sequence data include the phylogenetic uncertainty of reconstructing trees using biparentally inherited markers that may show evidence of hybridization, introgression, incomplete lineage sorting, or other potential challenges [93]. Another challenge that may hinder the usefulness of the nrITS region in Arales is that the sequences are difficult to obtain in some taxa, most notably the Lemnaceae lineage that apparently has an exceptionally firm secondary structure that resists molecular data acquisition methods [59].

**Figure 5:**
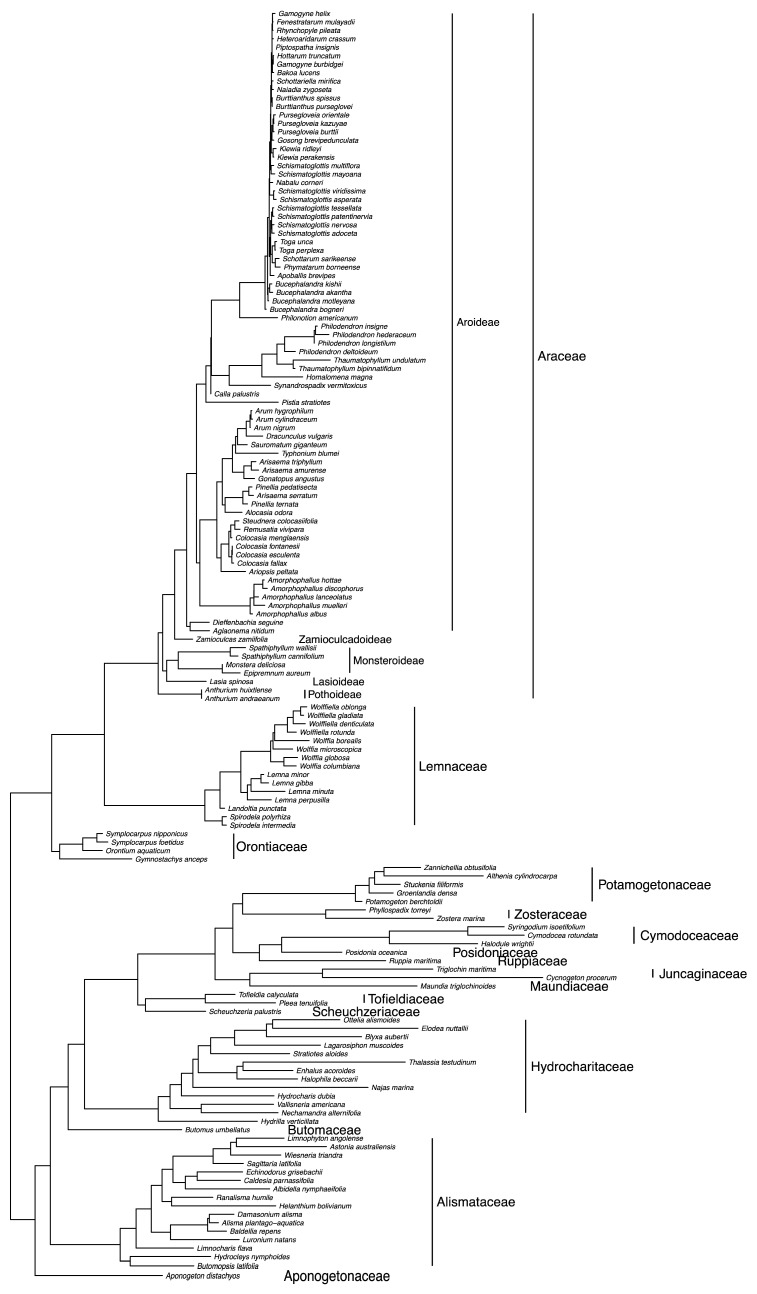
Phylogeny of Arales, constructed using combined DNA sequence data from nuclear ribosomal genes and spacers (18S and 5.8S rRNA, and the ITS-1 and ITS-2 spacers). The tree represents a maximum likelihood tree, constructed in IQ-TREE [18] using default settings.

We conducted an updated phylogenetic investigation using published and newly generated sequence data for the 18S ribosomal RNA gene and the nrITS region. Novel data were obtained using published methods [94,95,96,97], and GenBank accession numbers are provided in Supplementary Table S1. Prior to our study, there were very few 18S sequences available for Arales, with the exception of a complete sampling of Lemnaceae species [59]. We were able to add nuclear DNA data for 15 Arales species, including heretofore unavailable sequences for Araceae subfamily Zamioculcadoideae and other species spanning the breadth of diversity in Araceae s.s. The phylogenetic relationships that were determined using nuclear sequence data (Figure 5) recapitulate the same major relationships that are depicted on the plastid phylogeny (Figure 2 and Figure 4). Orontiaceae resolve as the sister to the clade containing Araceae s.s. and Lemnaceae, and the latter two families are reciprocally monophyletic.

Although they are useful for inferring nuclear DNA evolution, the nuclear ribosomal genes and internal transcribed spacer regions reflect the extremes of conserved and variable sequences, respectively. Efforts are ongoing to expand the number of nuclear gene regions that can be used for phylogenetic reconstruction, including the ambitious One Thousand Plant Transcriptomes Initiative [98]. However, the taxonomic sampling from Araceae s.l. remains limited for such an analysis. 

Targeted efforts are underway to increase the available nuclear sequence data for Lemnaceae [45], and it would be valuable to enact a parallel approach to studying other Arales and Alismatales taxa. Sequencing additional nuclear genes for taxa in Araceae s.s., Lemnaceae, and Orontiaceae may even further corroborate the phylogenetic distinctness of these lineages and potentially provide insights into their genome evolution.

### 2.3. Mitochondrial Molecular Data

Mitochondrial gene data are rarely used in angiosperm phylogenetic studies, but they potentially represent an independent source of phylogenetic data that are predominantly inherited uniparentally like in the plastid [99]. Some of the earliest attempts at reconstructing plant phylogenies with mitochondrial data used the *cox1* gene (cytochrome c oxidase subunit 1), a homolog of the most widely used phylogenetic marker in animals (where it is commonly known as COI) [100]. Unfortunately, the angiosperm *cox1* sequences were determined to have relatively few nucleotide polymorphisms in the coding region and a variable intron, the presence or absence of which is not phylogenetically informative in many taxa [101,102]. Because of this and the contrasting high utility of the plastid sequence data, mitochondrial genes were largely abandoned as phylogenetic markers. Although *cox1* turned out to be minimally useful, other mitochondrial genes have shown promise for recapitulating the broad topology of flowering plants [103,104]. Protein-coding and intron regions of four genes (*atp1*, *matR*, *nad5*, and *rps3*) are potentially effective for building an independent phylogenetic hypothesis about the evolution of angiosperms.

Preliminary phylogenetic analyses using mitochondrial genes [103,104] resolved a monophyletic Arales and also a larger clade that also included Alismataceae, Juncaginaceae, and Potamogetonaceae (i.e., Alismatales s.s.), as well as Tofieldiaceae (Tofieldiales). The Alismatales clade is characterized by curiously large branch lengths relative to comparable lineages (e.g., using the plastid phylogeny for reference), and considerable sequence data have been generated for this group to help illuminate the interesting evolutionary history of their mitochondrial genomes [105,106,107]. 

**Figure 6:**
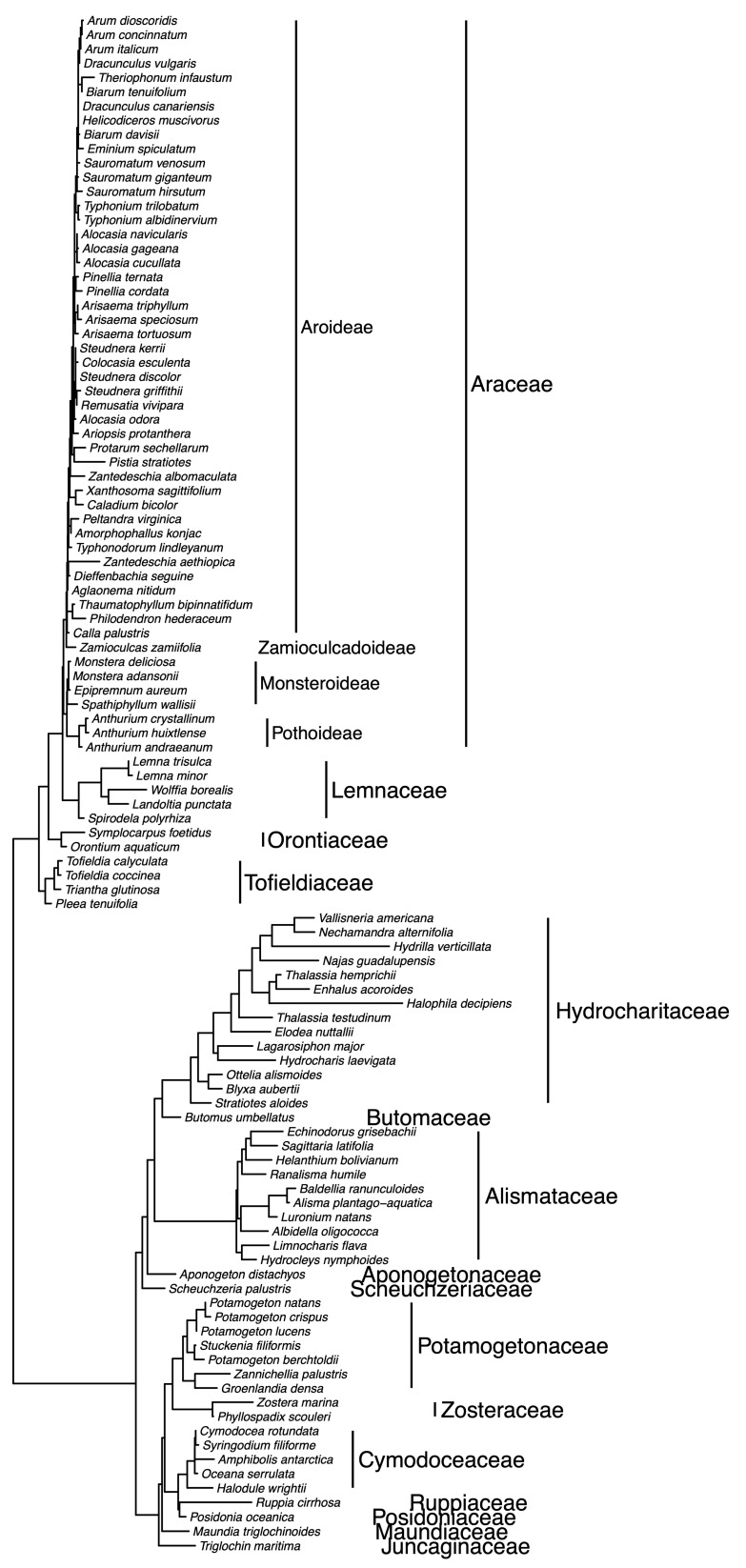
Phylogeny of Arales, constructed using combined DNA sequence data from four mitochondrial regions (*atp1*, *matR*, *rps3*, and *nad5*). The tree represents a maximum likelihood tree, constructed in IQ-TREE [18] using default settings.

Our updated phylogenetic analysis using expanded sampling of Arales s.l. taxa (Figure 6) produced a phylogeny that supported many of the same relationships as the plastid tree (Figure 2 and Figure 4). Thus, the mitochondrial data may become a useful complement to the organellar sequence data contained in the plastid. Mitochondrial genomes can be obtained using the same next-generation sequencing techniques that are enabling so many plastid genomes to be published [108]. A recently published mitochondrial genome for *Spirodela polyrhiza* (Lemnaceae) [109] may represent the beginning of a surge in similar data from other Arales species.

## 3. Morphological Data

The morphological distinctness of the aroids is undeniable. They have cells with calcium oxalate crystals, and a distinctive inflorescence type consisting of a typically showy bract (spathe) subtending a thick spike of tiny flowers (spadix) [1]. The inflorescence similarity between Araceae s.s. and Orontiaceae enabled them to be classified together, however, there are some exceptions to the idea that all aroids can be identified by the spathe + spadix inflorescence type. Firstly, two Orontiaceae genera, *Orontium* and *Gymnostachys*, lack spathes (Figure 1A,C), and the latter genus even exhibits a branched inflorescence (Figure 1C) unlike any known in Araceae s.s. Additionally, a distinctive inflorescence architecture, with continuation shoots produced in the penultimate leaf axil, characterizes nearly all the Araceae s.s. taxa, whereas this trait is absent from Orontiaceae [5]. The evolutionary origins of superficially similar organs are important to consider. In a phylogenetic context, the inflorescence spathe is reconstructed to be absent from the common ancestor of Orontiaceae, and thus its evolution at the root of Araceae s.s. is independent of the origin in *Lysichiton* and *Symplocarpus* [5]. Secondly, several more distantly related angiosperm groups have a spadix-like inflorescence, such as Acoraceae (Acorales), Cyclanthaceae Poit. ex A.Rich. (Pandanales R.Br. ex Bercht. and J.Presl), and even Piperaceae Giseke (Piperales Bercht. & J.Presl) [110]. Moreover, Hydrocharitaceae inflorescences are subtended by one or two bracts that also are termed ‘spathes’ [111]. Therefore, neither spathe nor spadix is unique to Araceae s.l.

The extreme morphological reduction that characterizes Lemnaceae has always made them an awkward fit for Araceae s.l., and their highly reduced morphology undermines the morphological characters that otherwise might unify plants in this group. If duckweeds are considered to belong to Araceae s.l., then the family must be characterized as having a distinctive inflorescence and vegetative features, except for the duckweed lineage that has no such features. If Lemnaceae and Orontiaceae instead are retained as separate from Araceae s.s., then each family can be identified readily by its distinctive features. Araceae s.s. and Lemnaceae each have a wealth of characteristics that unify their species, leaving only Orontiaceae as relatively difficult to classify.

The Orontiaceae clade includes two genera (*Lysichiton* and *Symplocarpus*) with inflorescences that are more like those found in Araceae s.s. (compare Figure 1B,D to Figure 1G–J), and two other genera (*Gymnostachys* and *Orontium*) that do not quite conform to the spathe+spadix morphology. Looking beyond the more obvious inflorescence features, there are in fact morphological traits that unite Orontiaceae and can be used to diagnose its constituent species from Araceae s.s. taxa. A morphological phylogenetic analysis of extant Arales [5] identified several features that are diagnostic or nearly so for Orontiaceae, including a collenchyma type [112] that is found only in *Lysichiton* and *Symplocarpus*, coincidentally the same two Orontiaceae genera whose inflorescence spathes otherwise make them appear superficially more similar to Araceae s.s. (petiole collenchyma is absent in *Gymnocarpus* and *Orontium*). Perhaps most noteworthy, there are leaf shape and venation patterns that can enable confident identification of Orontiaceae species, even in fossil material [113,114]. Thus, under more careful examination, there are more than a few characters that contradict the apparent similarity between Araceae s.s. and Orontiaceae.

## 4. Chromosome Number Evolution

Another superficial similarity that has been noted between Orontiaceae and Araceae s.s. is their range of chromosome numbers. Chromosome numbers have been reported for a large number of Arales taxa, including all genera of Lemnaceae and Orontiaceae. The reported base chromosome numbers for Orontiaceae (x = 12, 13, 14, or 15) have all been observed in Araceae s.s. genera, whereas Lemnaceae were reported to have a base x = 10 chromosome number that is nearly unique among other Arales [5]. However, the simplified x = 10 value fails to account for the wide variety of chromosome numbers that have been reported for Lemnaceae [115], many of which are not divisible by 10. An approach aimed specifically at reconstructing chromosome number evolution in Arales produced different numbers for the most recent common ancestors of Orontiaceae (*n* = 17), Lemnaceae (*n* = 22), and Araceae s.s. (*n* = 15) [13]. It should be noted, however, that these numbers were selected as the most likely among several competing values that also were highly probable. 

A more precise reconstruction of chromosome evolution requires evaluating the synteny of homologous chromosome regions. Cao et al. [62] developed a method to visualize syntenic chromosome regions across Lemnaceae species and inferred that seven ‘ancestral chromosome blocks’ later became duplicated and distributed across *n* = 20 chromosomes in Lemnaceae [62,115,116]. In Araceae s.s., the full genome sequence has been obtained for *Colocasia esculenta*, and a synteny analysis indicates *n* = 14 linkage groups [117]. Synteny analyses will be important for developing appropriate chromosome comparisons among Arales lineages and for reconstructing evolutionary changes. Fortunately, additional genome sequences are forthcoming, and these will enable a thorough comparison among species at every taxonomic level.

## 5. Biogeography

One final evolutionary aspect of comparison is that of biogeography or the ancient dispersal processes that are manifest in the geographic distributions of extant species. A thorough evaluation of ancestral biography has been conducted for Lemnaceae and determined that the ancestor of the family likely diversified in the Americas [15]. We applied a similar approach to the phylogeny for Arales, Alismatales, and Tofieldiales, using the ‘realm’ division of the terrestrial ecoregions of the world [118]. Ancestral distribution ranges were reconstructed for species that are represented on the plastid phylogeny, which has the most extensive taxon sampling in our study and also represents the phylogenetic topology that has been used in most other studies of Arales evolution [4,5]. Native ranges for species were obtained from the Kew Plants of the World Online database [119]. Ecological realm boundaries were approximated onto the geopolitical boundaries identified by the Taxonomic Databases Working Group [120], and membership in one or more realms was determined according to the inset map in Figure 2.

The biogeography analysis illustrates the temperate northern distribution of Orontiaceae taxa and reconstructs the ancestor of the family in North America or Eurasia (Figure 2). The temperate northern distribution of Orontiaceae and the generally northern distribution of Lemnaceae contrast with the decidedly tropical distribution of most Araceae s.s. species. Our analysis estimates that the common ancestor of Araceae s.s. diversified in South America or southeastern Eurasia, where many of the extant species are found today. A small number of Araceae s.s. genera have northern temperate species (e.g., *Arisaema* Mart., *Calla* L., *Peltandra* Raf.), but the vast majority are tropical. The dispersals to northern temperate habitats were independent, and they are scattered across the phylogeny (Figure 2). Thus, even geographic distributions can be useful for distinguishing Araceae s.s. from Orontiaceae, as the latter are almost entirely temperate and the former are relatively rare at temperate latitudes.

## 6. Discussion

### 6.1. Nomenclatural Stability and Utility

Modern botanical taxonomy aims to circumscribe natural groups that descended from common ancestors and, if possible, groups that are clearly differentiated using morphological or anatomical characters. Molecular phylogenetic data have been invaluable for identifying monophyletic groups, but these data are unable to prescribe the appropriate size of the clade that should constitute an order, family, or genus. To address this question, it is instructive to compare taxa at the same rank, while considering their respective ages and degrees of morphological divergence. The 122 Ma crown age of Araceae s.l. is among the oldest of any angiosperm family [14,16,121] and even falls at the older range of crown ages for angiosperm orders (27.1–128.9 Ma, $\;\bar{x}$ = 88.2 Ma) [20]. In contrast, the crown ages of Lemnaceae (73 Ma), Orontiaceae (96 Ma), and Araceae s.s. (97 Ma) (i.e., the family-level categories that we propose in this paper) would be more consistent with the ages of other angiosperm families (0.0–139.4 Ma, $\;\bar{x}$ = 45.1 Ma) [6,121]. As previously demonstrated, the morphological divergence of Lemnaceae is unmistakable [12,58], and the independence of Orontiaceae is supported by numerous morphological characteristics [31].

In the era when relationships inferred from molecular phylogenetic studies frequently differ from traditional taxonomy, in the interest of stability it can be valuable to make the fewest reassignments from traditional categories. Lemnaceae are a firmly established group that molecular data have validated as monophyletic. Even amateur botanists are familiar with duckweeds and their distinctive growth form, and a diverse assemblage of duckweed genera all fall cleanly within the Lemnaceae category. In addition to maintaining nomenclatural stability, augmenting the number of family-level categories enables more effective discussions about the defining traits of each family. In contrast, amalgamating a large amount of taxonomic and morphological diversity into one large Araceae s.l. family obscures the features that unify Araceae s.s. and prevents facile discussion of Lemnaceae and Orontiaceae.

### 6.2. The Nature of a Plant Family

The advent of molecular phylogenetics has caused many traditional taxonomic categories to be reorganized, as categories are widely expected to reflect monophyletic evolutionary lineages. Adherents to prior categories have understandably resisted some of the recent taxonomic changes, but in general, the categories are trending toward greater stability and an enriched evolutionary perspective. In some cases, a small number of taxa can be reassigned to maintain monophyletic categories, but for other groups, a large number of reassignments are required. Among the more noteworthy examples are the monocot order Asparagales Link, which decreased the number of included families by half in the time between the initial [10] and the most recent APG publication [66], and the eudicot family Scrophulariaceae Juss., where the majority of major lineages (at the rank of tribe) ended up being assigned to other families [122]. Changes in the latter group became necessary because the morphological similarity of some species was not consistent with their evolutionary relationships as supported by molecular evidence. Throughout the taxonomic upheaval that resulted from the age of molecular systematics, taxonomists have strived largely to retain categories that are informative (in terms of morphology), equivalent to other categories at the same rank, and consistent as much as possible with traditional taxonomic categories [66].

There is not a strictly defined set of criteria for determining the boundaries of a plant family that would take into account, for example, evolutionary age, degree of morphological or molecular divergence, or the number of subordinate taxa. The most general guideline seems to be that plant families should be roughly equivalent to one another in these aspects, so that one might develop a general sense of what constitutes a typical plant family. In many respects, the family-level categories advocated herein (i.e., Araceae s.s., Lemnaceae, and Orontiaceae) are more in line with the ‘typical’ plant family, when compared against the alternative Araceae s.l. classification scheme. When compared to Alismatales s.s. families and families across the angiosperms, our proposed family categories for Arales are more similar in evolutionary age, morphological divergence, and diagnosability. Moreover, the recommended categories require fewer reassignments of genera or species than the Araceae s.l. alternative. Therefore, we maintain that the categories of Araceae s.s., Lemnaceae, and Orontiaceae are more stable and more useful.

## 7. Conclusions

Classification schemes are necessarily subjective, but ideally, they strive to achieve consistency, clarity, and utility for botanists. The subsumption of Lemnaceae into Araceae s.l. effectively has removed a useful taxonomic category that duckweed biologists have used for many years, and a category that was among the most clearly defined of any angiosperm family. In contrast, we argue that there is less of a need to preserve the four Orontiaceae genera within Araceae s.l., as these plants already have been recognized as distinct in the ‘proto-Araceae’ category and surely merit their own taxonomic category at the family level. Classifications that relied heavily upon plastid molecular data are now bolstered by data from the nuclear and mitochondrial genomes, which support the same major evolutionary relationships. The categories of Araceae s.s., Lemnaceae, and Orontiaceae as proposed herein are informative and stable, and their usage will promote a better understanding of each respective group by professional and amateur botanists alike. 

## Data Availability

All sequences used for tree reconstruction are available from GenBank (https://www.ncbi.nlm.nih.gov/; accessed on 12 November 2021). The respective accession numbers can be found in Supplementary Table S1.

## Supplementary Materials

The following are available online at https://www.mdpi.com/article/10.3390/plants10122639/s1. Table S1.xlsx (Accessions used for tree reconstruction), Arales-cpDNA-alignment.nex (Sequence alignment of plastid DNA for Arales), Arales-mtDNA-alignment.nex (Sequence alignment of mitochondrial DNA for Arales), Arales-nrDNA-alignment.nex (Sequence alignment of nuclear DNA for Arales).
